# Sealing performance of sealing structure in a sediment sampler under ultra-high pressure

**DOI:** 10.1038/s41598-023-45728-6

**Published:** 2023-10-29

**Authors:** Shudong He, Youduo Peng, Sawei Qiu, Xuelin Du, Yongping Jin

**Affiliations:** 1 School of Intelligent Manufacturing and Mechanical Engineering, Hunan Institute of Technology, Hengyang, 421002 China; 2 National-Local Joint Engineering Laboratory of Marine Resources Exploration Equipment and Safety Technology, Hunan University of Science and Technology, Xiangtan, 411201 China

**Keywords:** Environmental sciences, Ocean sciences

## Abstract

As a key component of a sediment sampler designed for ultra-high pressures, the sealing structure determines whether pressure retention can be reliably achieved. This study constructed a finite element model to study sealing performance and reveal the sealing mechanism. The effects of the hardness and compression rate of O-ring as well as seawater pressure on the sealing performance were studied. The study showed that a self-tightening seal can be formed when the coefficient of friction on the sealing surface is less than or equal to 0.25. In addition, the maximum contact stress of the O-ring increased nearly linearly with increasing pressure, and it was larger than the corresponding pressure. However, with increasing pressure, the maximum Von-Mises stress initially increased rapidly, then tended to stabilize, and then continued to increase. Although increasing the hardness reduced the principal strain, the stress increased correspondingly. Within the compression rate range from 10 to 25%, the hardness of the O-ring had a greater impact on the contact pressure than the compression rate. In order to further verify that the finite element analysis was accurate, the sealing performance was tested, and the results showed that the seal was reliable and capable of sealing a deep-sea sampler.

## Introduction

In the exploration and utilization of deep-sea resources, obtaining pressure-retained samples that maintain in-situ conditions is vital. To do this, seals that can withstand the ultra-high pressure of the deep sea are critical, but they present an essential technical challenge that needs to be overcome. The loss of pressure can result in the dissolution of a sample’s gas phase, loss of components, decomposition of organic matter, and death of barotropic microorganisms. Due to the limitation of sealing technology, only about 0.01% of the total area of ocean depths have been sampled and studied by human beings^1^. Therefore, it is vital to research the sealing performance of sampler sealing structures under ultra-high pressures. There are many types of seals utilized in deep-sea sampling equipment, among which the O-shaped rubber seal is the most typical sealing structure. It is widely used because of its simple structure and reliable sealing capability, and because scholars have comprehensively examined its sealing performance.

Many studies have examined single seal structures. For example, Shuhua et al.^2^ investigated the effect of a baffle plate on sealing performance of the underwater glider in a deep water environment using a numerical method and concluded that the baffle plate improved the sealing performance of the O-ring. Qiao et al.^3^examined the effect of different metal material properties on sealing performance of O-ring by three-dimensional finite element analysis (FEA), which showed that material is an essential influencing factor in the sealing structure of the O-ring. Davies et al.^4^ tested a nitrile rubber joint for a deep-diving submersible. Chen et al.^5^conducted a finite element mechanical analysis of O-ring seals made of acrylonitrile-butadiene rubber to investigate the deformation of O-ring seals under oil pressure of 3 MPa, as well as the distribution of contact pressure and shear stresses between the shaft and the sealing contact surface. Zhou et al.^6^ concluded that the maximum contact pressure between the O-shaped rubber seal and the shaft increases with the increase of the seal compression rate and the working oil pressure by finite element analysis of the O-shaped rubber seal. Nikas^7^ applied the theory of Elasto-Hydrodynamic Lubrication using nonlinear finite element method to analyze stress distribution on a seal, examining the thickness of the oil film and leakage of rectangular seals under different working conditions. Abouel-Kasem^8^ used the finite element method to study stress and strain fields of six different elastic materials to analyze their leakage rates and to derive the expression for the leakage rate of elastic sealing materials over certain pressure and temperature ranges.

Although static analyses based on the finite element method can provide guidance for seal design, it is impossible to know how the seal will behave during the actual operation, not to mention to predict the leakage and friction generated during the sealing process. In order to better understand the reciprocating seal mechanism, many studies worldwide have developed lubrication analysis models in combination with engineering practices to explore the factors affecting sealing behavior and performance. Nikas^9^ established a theoretical model for seals with a retaining ring, studied the influence of parameters such as medium viscosity and surface roughness of the retaining ring on the sealing leakage under the condition of medium pressure between 1 and 35 MPa, and calculated the optimum roughness values of minimum leakage under different pressures. Salant et al.^10^ assumed that the piston rod surface is smooth to have established a mixed lubrication soft elastic flow model. The roughness effect is coupled in the model by modifying the oil film pressure distribution by flow factors and generating rough peak contact pressure. One study found that, in theory, a relatively small critical roughness value is required before a seal will meet the zero leakage requirement, and the critical value is affected by the operating conditions and the size of the seal structure. The friction surface cannot be absolutely smooth due to limitations in the processing technology. Therefore, Visscher^11^ studied how three different roughness values of a piston rod affect rectangular seal leakage and friction, finding that not only is the assumption of full-film lubrication conditions not applicable, but also that friction surface roughness will affect the leakage of the seal and the friction. Hirano^12^ found through experiments that, at the instant of startup or stroke transition (change of speed direction), the rubber of a seal will act viscous due to the rupture of the oil film between the contact interfaces. Further, Aston et al.^13^ studied the influence of thermal viscoelastic effect on seal deformation and friction, analyzed the stress relaxation and resilience characteristics of the seal at rest, and pointed out that high temperatures reduce the elastic modulus of the rubber, soften the rubber and reduce the friction. During the actual use of the combined seal, especially during the start-up phase or stroke transition, the seal behavior is transient due to the change in the reciprocating speeds.

The above theoretical research has produced important insights, however, few studies have examined seals in actual ultra-high-pressure environments. Kaneta^14^ was the first to study the transient elasto-hydrodynamic lubrication behavior of reciprocating sealing. Based on the assumption that the contact pressure at the seal interface is Gaussian parabolic, he put forward the necessary conditions for forming a full-film lubrication, that is, the stroke length is not less than twice the length of the contact area. Nikas^15^ took the combined seal with retaining rings as the research object. He established a transient elasto-hydrodynamic lubrication model with comprehensive consideration of the extrusion effect, roughness effect and thermal effect, investigated the influence of transient velocity and pressure on seal leakage and friction, and revealed the average films of seal contact interface under transient working conditions. Based on the hybrid lubrication model, Wang et al.^16^ investigated the coupling relationship between medium viscosity and seal surface roughness under variable- velocity conditions, and gave the critical roughness values of O-rings to meet the zero-leakage requirement under different medium viscosities. The above theoretical research has yielded fruitful results, however, there are few literature on the seals that are really applied in ultra-high-pressure environment.

To date, most relevant research efforts have focused on theoretical examinations of dynamic and static rubber seals, which have produced a variety of findings that have contributed to the analysis of sealing performance. However, most physical studies have analyzed the sealing performance of sealing structures under conventional pressures (i.e., low, medium, and high), and few have focused on examining combined sealing technologies under ultra-high-pressure conditions (as high as 115 MPa). Therefore, it is necessary to research the performance of the sealing structures of samplers under ultra-high pressures. In this paper, first we describe the self-tightening sealing mechanism of the rubber sealing ring and the structure of the combined seal under ultra-high-pressure conditions, and subsequently, we establish the numerical calculation model for the combined sealing structure of the rubber O-ring under ultra-high pressures. Using the model, the preconditions for forming self-tightening sealing were deduced. Based on the established numerical model, the influence of sealing structure parameters, seawater pressure, and initial compression rate on the seal contact stress and O-ring stress, as well as the sealing performance, were investigated in detail. This analysis was then used to reveal the sealing mechanism and failure mechanism of the ultra-high-pressure combined seal, providing a scientific basis for the reasonable design of the structure and parameters of ultra-high-pressure combined seals.

## Mechanism of self-tight sealing

**Figure 1:**
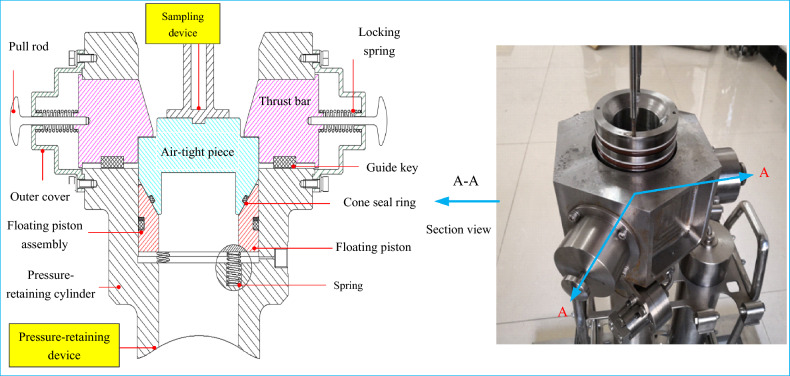
Schematic diagram of the self-tight sealing structure.

The sealing structure of the sampler is shown in Fig. 1. The sealing system adopted the conical surface plus O-ring sealing structure combination in the sealing ring structure. The floating piston and pressure-retaining cylinder adopted the O-ring plus retaining ring structure. The sampling device and floating piston used the conical surface plus fluororubber O-ring sealing structure. When the sampler reaches the seabed, the mechanical arm grabs the handle of the sampling device to make it inserted into the sediment, then slowly pulls out of the sediment and puts it into the pressure-retaining device. Under the action of the locking and support spring, the pressure-retaining device is sealed with the sampling device.

**Figure 2:**
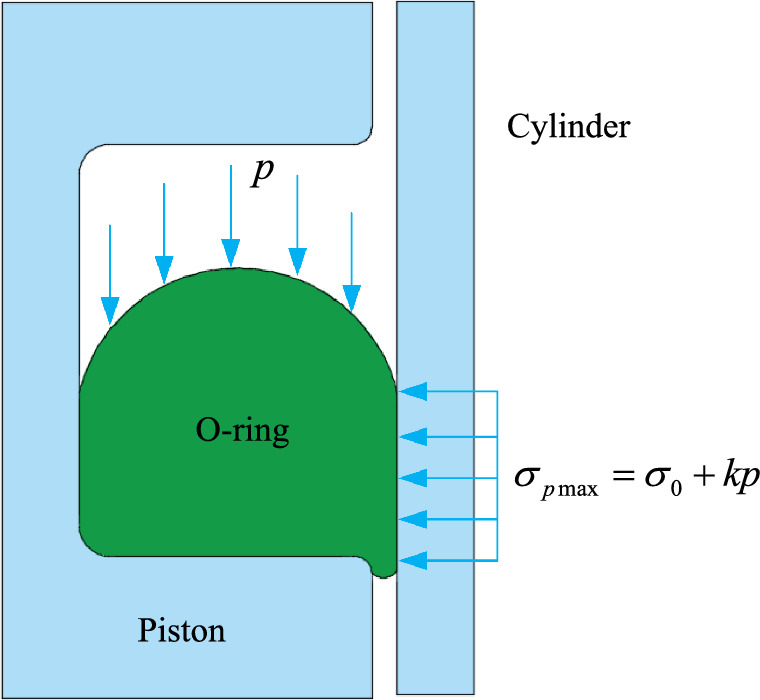
Schematic diagram of Sealing.

The self-tightening sealing of O-rings mainly depends on the incompressibility of the elastomer material and the initial pre-compression load. In the free state, the sealing ring is not constrained by external loads. The assembling of the seal between the cylinder and the piston sealing groove is realized when the cylinder pre-compresses the seal in a radial downward direction, as shown in Fig. 2. After assembling, the sealing ring is in a compressed state and the contact stress on the contact surface is σ_0_, thus the initial seal is affected. After pressurization, the medium in the groove only plays a role on one side of the seal. When the medium pressure *p* is high, the seal is pushed to the right side of the groove, at which time the contact stress on the seal contact surface is increased from the initial σ_0_ to $\sigma_{p\max }$.Thus, the sealing effect of the O-ring is further enhanced. After decompression, after returning to the initial contact stress σ_0_, the O-ring still maintains the seal, which is referred to as the self-tightening sealing mechanism. In simple terms, the necessary condition for sealing is that the contact stress on the sealing contact surface of the O-ring is not less than the sealing pressure:

1 $$ p \le {\text{min}}(\sigma_{0} ,\sigma_{p\max } {)} $$

2 $$ \sigma_{p\max } = \sigma_{0} + kp $$

where *p* is the seawater pressure, σ_0_ is the initial contact stress when the O-ring is installed, and *k* is the pressure coefficient whose value is close to 1 and its specific value is related to the hardness and elastic modulus of the O-ring.

## Ultra-high-pressure sealing performance

In order to ensure that the sampler to achieve a reliable sealing in the ultra-high pressure environment, this paper employs the finite element analysis method to study the sealing performance of the proposed ultra-high-pressure sealing structure, providing a scientific basis for the reasonable determination of ultra-high-pressure sealing structure and parameters.

### Numerical model

The three-parameter Mooney-Rivlin model was adopted for the rubber structure. This model fully complies with the characteristics of the large deformation and nonlinearity of rubber materials. Mooney’s theory is based on the following assumptions: (1) the rubber is isotropic and incompressible before deformation; (2) the Poisson’s ratio and modulus of elasticity are known; (3) the compression load of the sealing ring is caused by the displacement generated by the constraint boundaries; and (4) temperature does not have any effect on the material properties. Rivlin^17^ considered that the strain sealing function* W* is symmetric with respect to the three s. The three s are λ_3_, λ_2_, and λ_3_. Meanwhile, the three basic strain functions *I*_1_, *I*_2_, and *I*_3_ denote the length, area, and volume, respectively, which are expressed by:

3 $$ \left\{ \begin{gathered} I_{1} = \lambda_{1}^{2} + \lambda_{2}^{2} + \lambda_{3}^{2} \hfill \\ I_{2} = \lambda_{1}^{2} \lambda_{2}^{2} \lambda_{3}^{2} + \lambda_{2}^{2} \lambda_{3}^{2} + \lambda_{1}^{2} \lambda_{3}^{2} \hfill \\ I_{3} = \lambda_{1}^{2} \lambda_{2}^{2} \lambda_{3}^{2} \hfill \\ \end{gathered} \right. $$

where *I*_1_, *I*_2_ and *I*_3_are the three invariants of the deformation tensor; and λ_1_,λ_2_ and λ_3_are the three principal elongation ratios that satisfy:

4 $$ \lambda_{i} = 1 + \varepsilon_{i} $$

where $\varepsilon_{i}$ is the strain in the direction of the main axis.

According to the incompressibility of rubber material, the following relationship holds:

5 $$ I_{3} = \lambda_{1}^{2} \lambda_{2}^{2} \lambda_{3}^{2} = 1 $$

On this basis, Rivlin derived strain energy function *W* applicable to incompressible rubber materials.

6 $$ W = \sum\limits_{i + j = 1}^{N} {C_{{{\text{ij}}}} \left( {I_{1} - 3} \right)^{i} \left( {I_{2} - 3} \right)^{j} } $$

The following is obtained if the three terms *C*_10_, *C*_20_ and *C*_01_ are taken and *C*_ij_ = 0:

7 $$ W = C_{10} \left( {I_{1} - 3} \right) + C_{20} \left( {I_{1} - 3} \right)^{2} + C_{01} \left( {I_{2} - 3} \right) $$

The relationship between the principal stress and the principal elongation ratio of the rubber material is^18^.

8 $$ \sigma_{i} = 2\lambda_{i}^{2} \frac{\partial W}{{\partial I_{1} }} - \frac{2}{{\lambda_{i}^{2} }}\frac{\partial W}{{\partial I_{2} }} - \sigma_{h} ,i = 1,2,3 $$

where *σ*_*h*_ is the hydrostatic stress, which is the average of the three principal stresses. The following relationship exists between the three principal stresses:

9 $$ \left\{ \begin{gathered} \sigma_{1} - \sigma_{2} = 2\left( {\lambda_{1}^{2} - \lambda_{2}^{2} } \right)\left( {\frac{\partial W}{{\partial I_{1} }} + \frac{1}{{\lambda_{1}^{2} \lambda_{2}^{2} }}\frac{\partial W}{{\partial I_{2} }}} \right) \hfill \\ \sigma_{2} - \sigma_{3} = 2\left( {\lambda_{2}^{2} - \lambda_{3}^{2} } \right)\left( {\frac{\partial W}{{\partial I_{1} }} + \frac{1}{{\lambda_{2}^{2} \lambda_{3}^{2} }}\frac{\partial W}{{\partial I_{2} }}} \right) \hfill \\ \sigma_{3} - \sigma_{1} = 2\left( {\lambda_{3}^{2} - \lambda_{1}^{2} } \right)\left( {\frac{\partial W}{{\partial I_{1} }} + \frac{1}{{\lambda_{1}^{2} \lambda_{3}^{2} }}\frac{\partial W}{{\partial I_{2} }}} \right) \hfill \\ \end{gathered} \right. $$

For rubber tensile or compression experiments, where σ_2_ = σ_3_ = 0 is substituted into the above formula to get $\lambda_{2}^{2} = \lambda_{3}^{2}$, joint formula (5) can be obtained:

10 $$ \sigma_{1} = \left( {2\lambda_{1}^{2} - \frac{2}{{\lambda_{1}^{{}} }}} \right)\left( {\frac{\partial W}{{\partial I_{1} }} + \frac{1}{{\lambda_{1}^{{}} }}\frac{\partial W}{{\partial I_{2} }}} \right) $$

By substituting formulas (4), (7) into the above equation, the stress–strain relation for the 3-parameter Mooney-Rivlin model can be obtained:

11 $$ \sigma = \left( {2\left( {1 + \varepsilon } \right) - \frac{2}{{\left( {1 + \varepsilon } \right)^{{_{2} }} }}} \right)\left( {C_{10} + 2C_{{^{20} }} \left( {\left( {1 + \varepsilon } \right)^{2} + \frac{2}{1 + \varepsilon } - 3} \right) + \frac{{C_{01} }}{1 + \varepsilon }} \right) $$

### Finite element model

**Figure 3:**
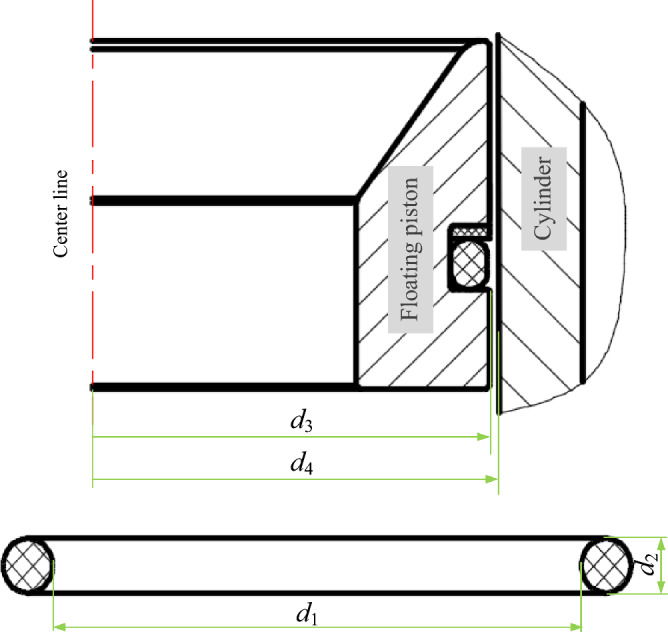
Geometric model.

To analyze the O-ring, a geometric model of the sealing system, including components such as the floating piston, sealing ring, and cylinder is established. The geometric model is shown in Fig. 3, should be established first. The sealing structure is symmetric about its centerline, so the radial loads on the O-ring are axisymmetric. In order to facilitate analysis and calculation, a two-dimensional axisymmetric model of the sealing structure is established. The model of the sealing structure and the related sizes are shown in Fig. 3. The diameter of the O-ring (Φ) is 5.3 mm, the width of the sealing groove (b) is 7.1 mm, the depth (h) is 5 mm, the clearance between the floating piston and the cylinder (δ) is 0.01 mm, the cylinder round angle (r_1_) is 0.5, the groove bottom chamfer radius (r_2_) is 0.5 mm, and the groove prism chamfer radius (r_3_) is 0.2 mm. Due to the hardness of the floating piston and the cylinder, both are considered to be rigid bodies, with moduli of elasticity of E_0_ = 210 GPa and Poisson’s ratios of μ_0_ = 0.3. The sealing piece is considered to be a flexible body, with Poisson’s ratio of 0.499 and a modulus of elasticity determined according to the hardness of the selected fluororubber.

**Figure 4:**
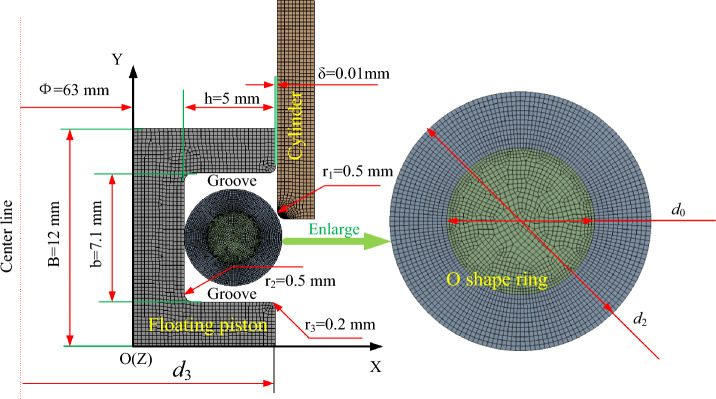
Finite element calculation model.

In this paper, the finite element analysis model of sealing is established using ANSYS software. The groove, the cylinder, and the O-ring are meshed with a quadrilateral mesh. At the same time, the model is divided into regions (inner circle and outer ring) with different mesh sizes. In order to minimize the influence of mesh distortion on the convergence and accuracy of the calculation, mesh adaptive redrawing technology is used in the sealed contact area in the outer ring of the sealing circle which experiences large deformation. This allows the mesh with large deformation to move independently in the calculation without affecting the overall calculation accuracy. After debugging and grid verification analysis, the finite element calculation model of the sealing structure is finally determined, as shown in Fig. 4.

Common contact algorithms include penalty function algorithm, conventional Lagrange algorithm, incremental Lagrange algorithm. Due to the large deformation and non-linearity of the sealing ring and the characteristics of calculation are not easy to converge, the penalty function contact algorithm with good convergence, high solving efficiency and strong adaptability to over-constraints is adopted in this paper^19^. The contact surface is sliding friction, and the Coulomb friction type is used to define the friction behavior between contact surfaces^20^.These include the contact interactions between the O-ring and the inner wall of the cylinder and between the O-ring and the groove. Full constraints are applied to the groove, i.e., the displacements of the groove in the X, Y, and Z directions are set to 0. Two load steps are used to realize loading in the simulation process:

- Step 1: Installation and pre-compression of O-ring seals. First, a fixed boundary constraint is applied to the groove, and then the O-ring is pre-compressed by applying a negative Y-axis displacement to the cylinder to force it move slowly and linearly to the expected installation position, effectively simulating the installation process.
- Step 2: Seawater pressurization process. Different seawater pressures are applied to the upper side of the O-ring and the seawater contact unit to simulate the sliding of the O-ring from the middle of the groove to the other side until the final ultra-high working pressure is reached.

## Results and discussion

### Analysis of sealing mechanism

**Figure 5:**
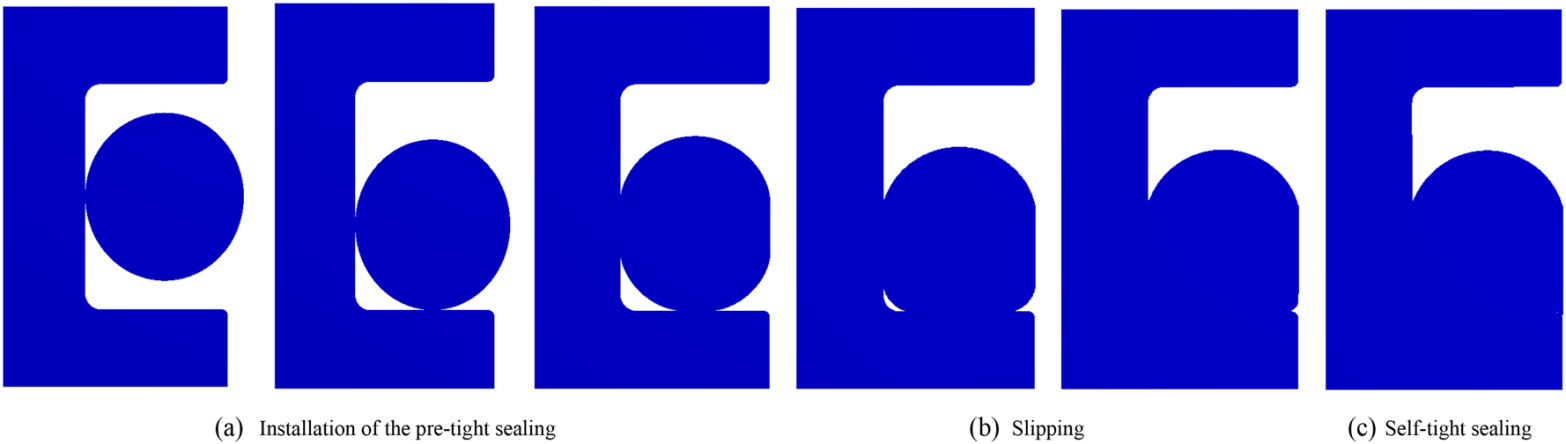
Three stages of the sealing process.

The results of the numerical model of deep-sea ultra-high-pressure sealing are shown in Fig. 5. The sealing process mainly includes three stages: the installation of the pre-tight sealing, slipping, and the self-tight sealing.

**Figure 6:**
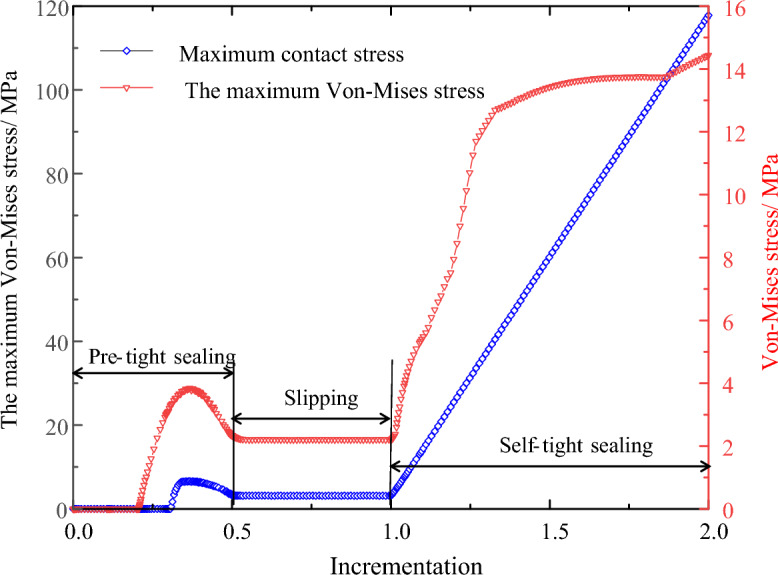
Stress change in sealing process (with a compression rate of 15%, seawater pressures of 115 MPa and material hardness of 85HA).

The installation and pre-tightening of the seal refers to the process in which the O-ring seal is pre-compressed by an external force acting on the O-ring. In this process, the O-ring generates contact stress on the contact surfaces of the grooves and the cylinder wall, referred to as pre-tightening of the sealing piece, as shown in Fig. 5a. As the seawater pressure starts to act on the O-ring seal, increasing from zero, friction *f*_p_ is generated between the O-ring and the sealing groove and the cylinder wall, which forces the O-ring to resist the seawater pressure. This process is key to the establishment of the initial seal. To achieve an effective initial seal, the maximum contact stress on the contact surface under the pre-compression load must not be less than the seawater pressure. During this process, the maximum contact stress and maximum Von-Mises stress of the O-ring undergo changes, as shown in Fig. 6.

When the pressure of the seawater pressure is below the critical value (*P*_f_), that is, when the axial force generated by the seawater pressure on the O-ring is not greater than the friction, the O-ring is kept stationary by the friction. Based on a force analysis of the O-ring seal, according to the condition that the axial force is equal to the friction force, the critical pressure required for sealing can be obtained as follows:

12 $$ P_{{\text{f}}} = \frac{{4f_{p} }}{{{\pi (}d_{1}^{2} - d_{2}^{2} )}} $$

The value of friction can be expressed as:

13 $$ f_{p} = 2{\uppi }\int_{0}^{b} {f_{\tau } D_{f} \sigma_{0} (x)} dx $$

Under pre-compression load, the contact width *b* between the O-ring and the cylinder depends on the initial compression rate of the O-ring *ε*_0_ and the initial section diameter *d*_3_, the value of which can be obtained by equation^19^:

14 $$ b = 3\varepsilon_{0} d_{3} $$

where, *ε*_0_ can be calculated by the following formula:

15 $$ \varepsilon_{0} = \frac{{d_{1} - d_{2} }}{{d_{3} }} $$

In these two equations, *σ*_0_(*x*) is the contact stress distribution function for contact width (*b*) of the O-ring;* D*_*f*_ is the diameter of the contact surface; *d*_1_, *d*_2_, and *d*_3_ are the diameters of the groove, cylinder, and O-ring, respectively; and *ε*_0_ is the initial compression rate, *f*_τ_ is the friction coefficient, *f*_*p*_ is the friction, which can be approximated as the following formula^18^:

16 $$ f_{p} = 2{\uppi }bf_{\tau } \cdot \frac{{\sigma_{0\max } }}{1.5} \cdot \left( {\frac{{d_{1} + d_{2} }}{2}} \right) $$

Formulas (12), (14), (15) and (16) are combined to obtained the following formula:

17 $$ P_{{\text{f}}} = 4f_{p} \sigma_{0\max } $$

As the seawater pressure increases, it will eventually reach the critical value *P*_f_ when the axial force on the O-ring exceeds the friction on the contact surface. At this point, the O-ring will overcome the frictional resistance on the sealing surface and slide in the direction of increasing seawater pressure, as shown in Fig. 5b. During the O-ring slipping process, the maximum contact stress and maximum Mises stress remain basically unchanged.

When the O-ring reaches the lower end of the groove, it makes contact with the lower end of the groove, which exerts a reaction force against the O-ring. When the sum of the reaction force and friction are equal or greater than the axial force of the seawater pressure on the O-ring, the sliding process ends and the self-tightening sealing process begins. As the seawater pressure continues to increase, the contact stress on the sealing surface also increases, and the O-ring is gradually forced to fill the corner space at the lower end of the sealing groove, as shown in Fig. 5c. Taking the full ocean depth pressure as an example, when the maximum seawater pressure of 115 MPa is reached, the contact stress and the Von-Mises stress reach their maximum values of 117.83 MPa and 14.44 MPa, respectively, as shown in Fig. 6.

Based on the analysis of the above process, it can be seen that the O-ring during the pre-tightening and slipping processes was unable to achieve the self-tightening sealing function. The sealing contact stress only comes from the contact stress generated by the O-ring during the pre-tightening process, which does not increase with increasing seawater pressure, which is how self-sealing is realized. Therefore, in order to prevent leakage before self-sealing, the maximum contact stress (*σ*_0max_) under pre-compression load must be equal to or greater than the critical pressure (*P*_f_) required for the O-ring to slip. Thus, the following can be obtained:

18 $$ \sigma_{0\max } \ge P_{{\text{f}}} = 4f_{p} \sigma_{0\max } $$

and

19 $$ f_{{\text{t}}} \le \, 0.{25} $$

Therefore, in order to realize effective sealing, it is necessary to reduce the roughness of the sealing groove and O-ring to ensure that the coefficient of friction *f*_τ_ is less than or equal to 0.25. An alternative method, which is both more direct and effective, is to put the O-ring in direct contact with the lower end of the groove during installation, so that the sealing structure is already in the self-tightening stage before being subjected to the pressure of the medium. Specifically, during the installation of the floating piston, the retaining ring should installed in the direction of the seawater pressure. At the same time, the floating piston is assembled downward towards the cylinder to ensure that the O-ring is at the end position upon completion of the installation and immediately establishes the self-sealing function.

### Influencing factors

The reliability of the sealing structure is evaluated based on two criteria^21–24^: first, whether the maximum contact stress between the O-ring and the groove is greater than the seawater pressure. Secondly, whether the O-ring will crack during the working process, thus resulting in sealing failure. Generally, crack-prone areas tend to have higher Von-Mises stress values. Based on these criteria, the following section examines sealing reliability by assessing the maximum contact stress and Von-Mises stress of the O-ring to reveal how they are influenced by three key factors: seawater pressure, compression rate, and material hardness.

#### Hardness of rubber material

Two material parameters, i.e., C_10_ and C_01_, of the Mooney–Rivlin model are used to characterize the rubber material. Usually, the hardness value of rubber (HS) can range from 60 to 90 HA. In general, the larger the external pressure is, the higher the hardness value of the selected rubber must be. Therefore, O-rings with material hardness values of 75 HA, 80 HA, 85 HA, 90 HA, and 95 HA were taken as examples; their physical properties are shown in Table 1.

**Table 1:** Material parameters under different hardness.

Hardness	75HA	80HA	85HA	90HA	95HA
*E0*	7.08	9.39	13.23	20.93	44.00
*C10*	0.94	1.25	1.78	2.79	5.87
*C01*	0.24	0.31	0.45	0.70	1.47

When considering the O-ring seal, usually the primary concern is the size of the seal. However, there is another factor that has a direct impact on deformation and maximum contact stress, the hardness of the O-ring. Softer O-rings (i.e., low hardness) facilitate easier installation, but are prone to falling off and being damaged, they can also be easily to be extruded under pressure, which will lead to seal failure. If the O-ring is too hard, installation can be inconvenient and the Von-Mises stress concentration will be intensified. Therefore, it is necessary to study the impact of O-ring material hardness on sealing performance by determining the distribution of the material deformation, maximum contact stress, and Von-Mises stress of O-rings with different material hardness values. This investigation will serve as an important design reference for the selection of O-ring material hardness.

**Figure 7:**
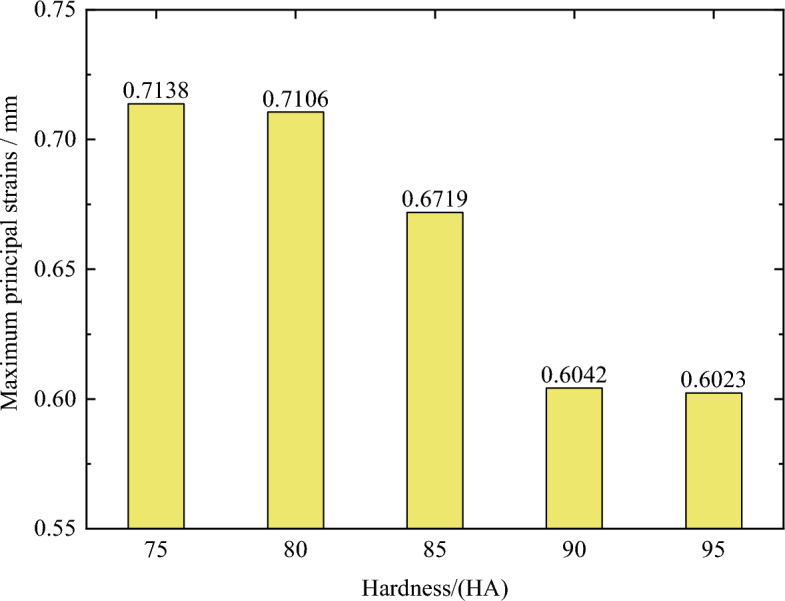
Maximum principal strain of O-shaped rubber under different material hardness (with a compression rate of 10% and seawater pressures of 115 MPa).

Figure 7 shows the distribution of maximum principal strain with a compression rate of 10% and O-rings with material hardness values of 75 HA, 80 HA, 85 HA, 90 HA, and 95 HA. It can be seen that the maximum principal strain decreased gradually with increasing hardness. When the material hardness increased from 85 to 90 HA, the maximum principal strain decreased by as much as 10.08%. However, when the material hardness increased from 90 to 95 HA, the maximum principal strain decreased by only 0.3%. Therefore, while there is a correlation between material hardness and deformation, it is not linear. Clearly, during the selection of seal materials, the influence of the material hardness on deformation and strain should first be taken into account.

**Figure 8:**
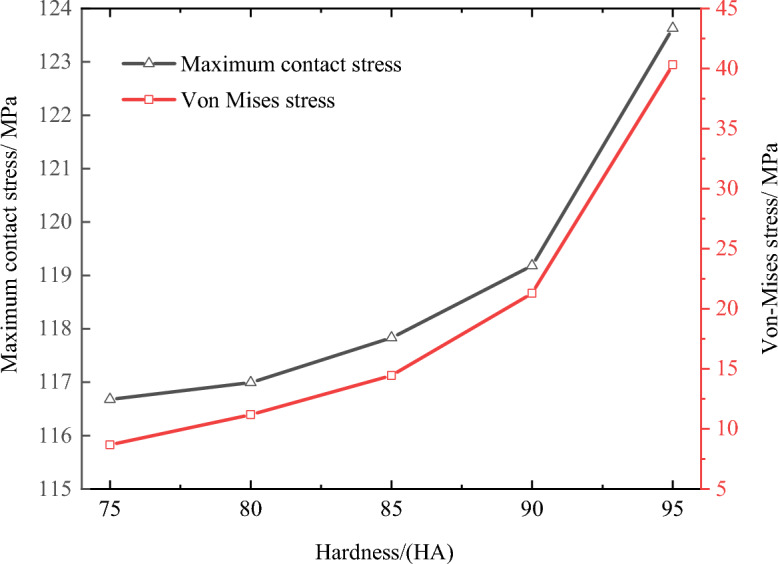
Maximum contact stress and Von-Mises stress of O-shaped rubber under different material hardness (with a compression rate of 10% and seawater pressures of 115 MPa).

**Figure 9:**
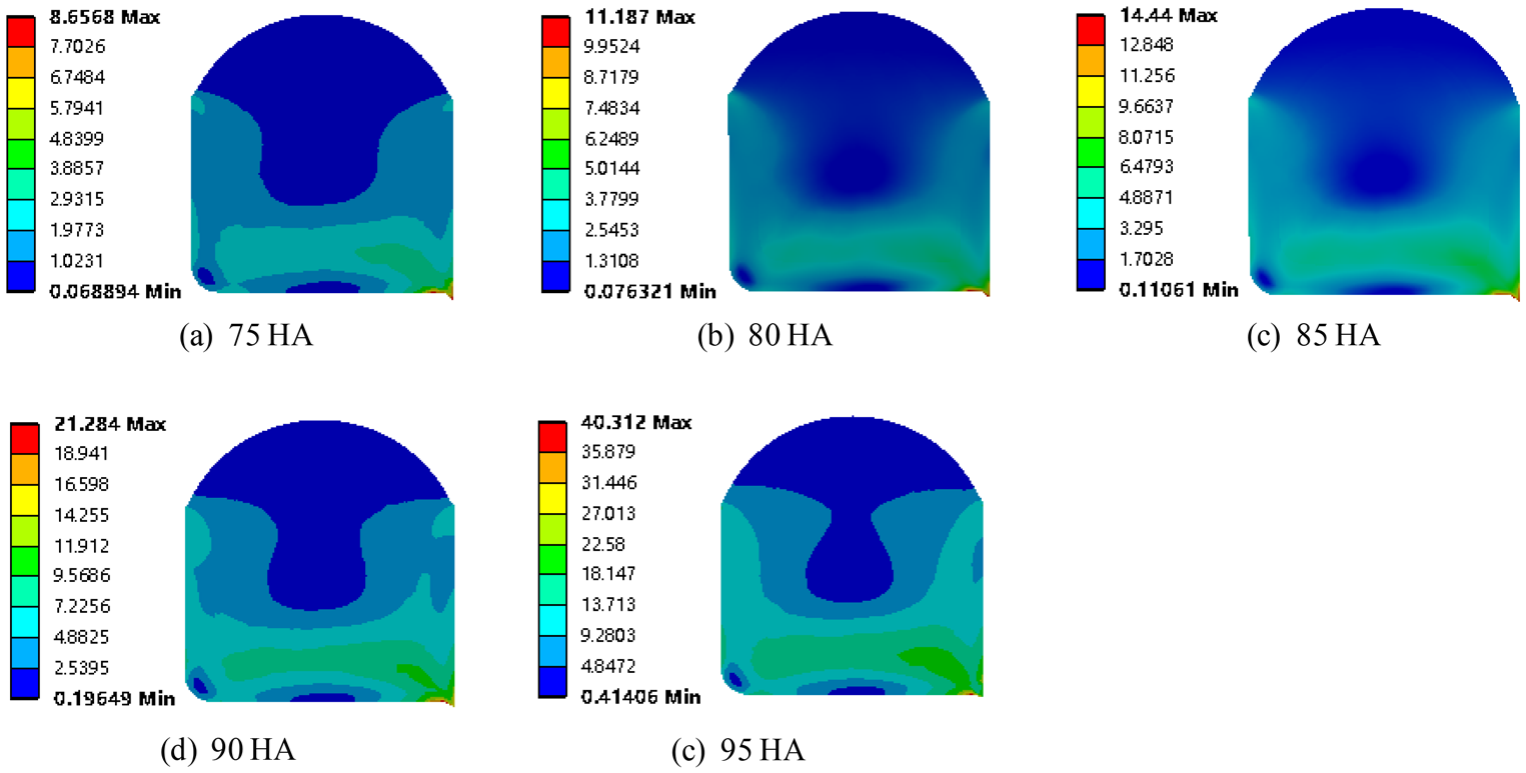
Maximum Von-Mises stress distribution of O-shaped rubber under different hardness (with a compression rate of 10% and seawater pressures of 115 MPa).

It is mentioned above that while a material with high hardness can reduce the maximum principal strain and ensure sufficient sealing contact stress to effectively prevent the seal material from being torn by tension. However, as the hardness increases, the maximum Von-Mises stress also increases. The stress distribution under different hardness conditions shown in Figs. 7, 8 and 9 just explains this phenomenon well. For example, when the hardness of the material is 90 HA and below, the maximum Von-Mises stress is within 21 MPa, but when the hardness of the material is 90 HA, the maximum Von-Mises stress occurs at the prismatic circle at the lower end of the groove, and the peak value is as high as 21.3 MPa, which exceeds the extension strength of the material which causes stress concentration and permanent deformation of the seal, resulting in sealing failure. Therefore, the material hardness of the O-ring seal should be below 90 HA, in which way the deformation, contact stress and Von-Mises stress are all taken into account.

#### Seawater pressure

The maximum working pressure of the sediment sampler is rated at 115 MPa, but this does not mean that the working pressure is 115 MPa every time. The actual working pressure is determined by the actual working water depth, so the working pressure will not always be the same. Therefore, it is necessary to study the sealing performance of the sealing structure under different pressure conditions.

**Figure 10:**
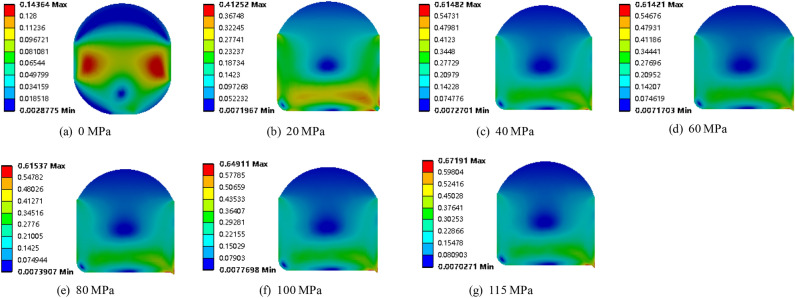
Maximum principal strain of O-shaped rubber under different pressure conditions (with a compression rate of 10% and material hardness of 85HA).

Cloud diagrams of the maximum principal strain distributions of the O-ring seals at sea water pressures of 0, 20, 40, 60, 80, 100, and 115 MPa, with pre-compression ratios of 10%, are shown in Fig. 10. Obviously, with increasing pressure, the clearance between the O-ring and the lower end of the groove was gradually reduced. When the pressure reached 20 MPa, the O-ring almost entirely filled the corner at the lower end of the groove. When the pressure reached 40 MPa, the sealing ring filled the clearance between the groove and the cylinder. In addition, the maximum principal strain increased with increasing pressure, reaching its maximum strain value of 0.672 mm at 115 MPa.

**Figure 11:**
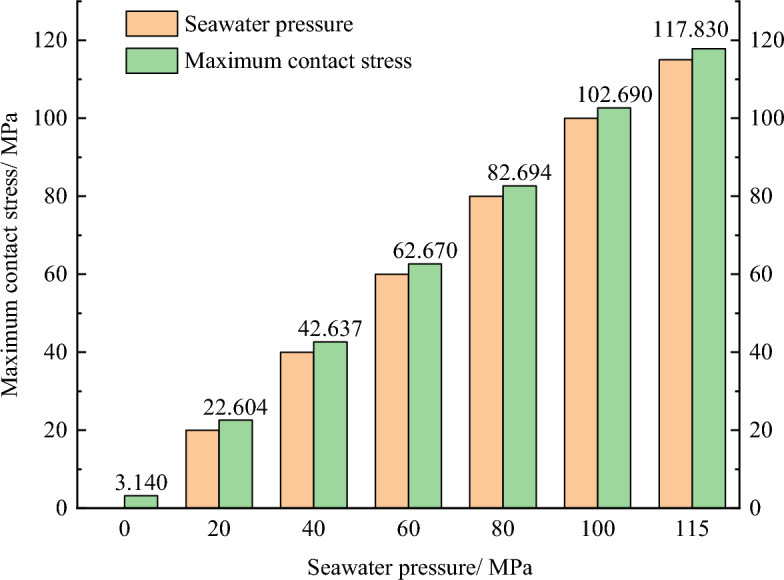
Maximum contact stress of O-shaped rubber under different pressure conditions (with a compression rate of 10% and material hardness of 85HA).

**Figure 12:**
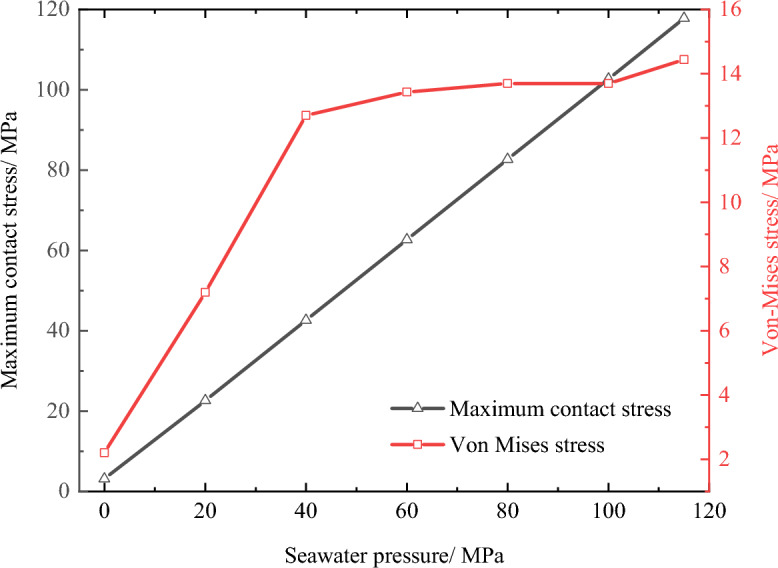
Maximum contact stress of O-shaped rubber under different pressure conditions (with a compression rate of 10% and material hardness of 85HA).

Figures 11 and 12 show the maximum contact stress distribution of O-ring seals under seawater pressures of 0, 20, 40, 60, 80, 100, and 115 MPa, respectively. It can be seen that the maximum contact stress of the O-ring increased with increasing seawater pressure, and all of the contact stresses were larger than the corresponding seawater pressures, indicating that the sealing requirements were met. In addition, Fig. 11 shows that the maximum contact stress of the O-ring increased linearly with increasing seawater pressure, which reflected the cumulative property of pressure transfer and precisely verified that the sealing mechanism of the O-ring was accurately characterized by Eq. (2).

**Figure 13:**
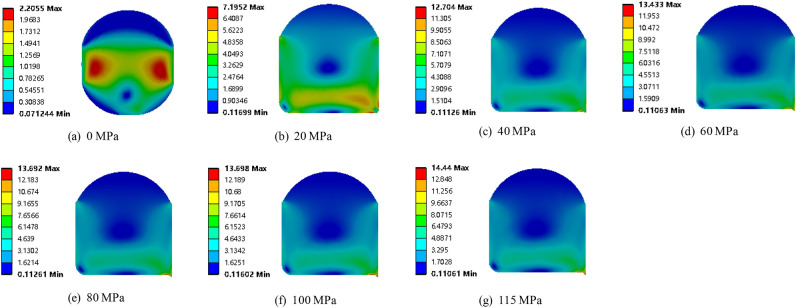
Von-Mises stress of O-shaped rubber under different pressure conditions (with a compression rate of 10% and material hardness of 85HA).

Figure 13 shows the Von-Mises stress of the O-shaped rubber seal under different seawater pressures. Clearly, when the seal was only subjected to the pre-compression load (seawater pressure is 0 MPa), the stress was concentrated on both sides of the middle position and distributed symmetrically. As the seawater pressure increased, the maximum Von-Mises stress increased and the O-ring shifted to the lower part of the groove.

The red area in Fig. 13 shows the area experiencing high stress. This area decreased with increasing seawater pressure, indicating that the stress was becoming more concentrated as the seawater pressure increased. When the seawater pressure exceeded 100 MPa, the concentration of stress experienced by the seal intensified at the lower right corner of the groove. Therefore, when entering ultra-high-pressure conditions, the O-ring seal will become prone to wear and tear, and this area will act as the contact area between the round prism at the lower end of the groove and the cylinder wall. In addition, even under the seawater pressure of 115 MPa, the maximum Von-Mises stress of the O-ring (about 14.44 MPa) remained below the extension strength of the O-ring (about 30 MPa), indicating it met the seal requirements.

#### Initial compression rate

Compression rate refers to the change in the cross-sectional diameter of the O-ring during initial installation. A high compression rate can achieve a high contact pressure, but too high a compression rate can increase the sliding friction and result in permanent deformation. Too small a compression rate, on the other hand, may cause leakage because the contact pressure does not meet the requirements. In general, the compression rate of a static seal is greater than that of a dynamic seal, but its highest value should still remain less than 25%. In order to facilitate the research, this paper examined O-ring compression rates ranging from 10 to 25%

**Figure 14:**
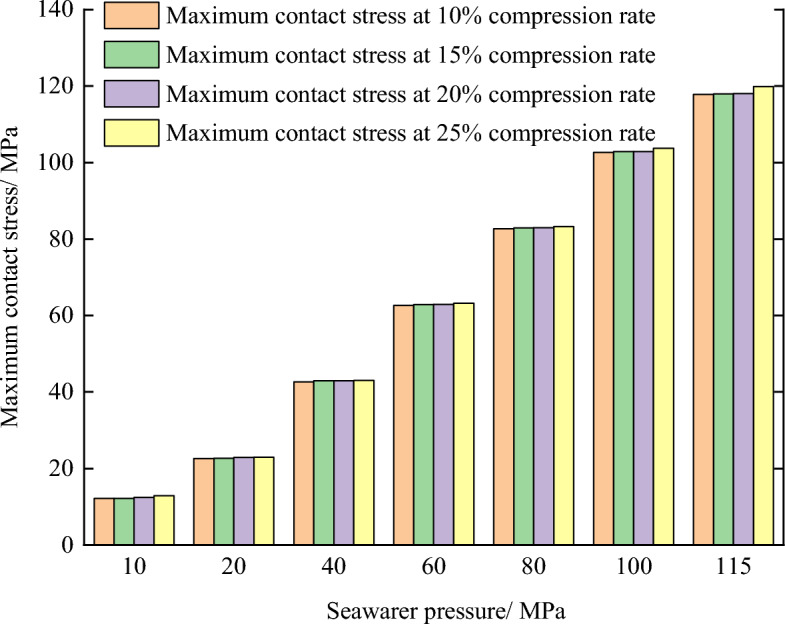
Maximumcontact stress of O-shaped rubber under different pressure conditions (with a material hardness of 85HA).

According to the sealing principle shown in Eq. (2), the maximum contact stress on the sealing surface is the sum of the initial contact stress and the approximate seawater pressure. It can be inferred that the maximum contact stress increases with increase in the compression rate under a certain seawater pressure range. Figure 14 shows how the maximum contact stress of the O-ring changed with the seawater pressure under different compression rates, which clearly verified the above-mentioned inference. When the seawater pressure is below 60 MPa, the maximum contact stress increases very little with the increase in compression rate, but when the pressure is greater than 60 MPa, the increasing trend is pronounced. For example, when the seawater pressure is 60 MPa, the maximum contact stress increases from 62.7 MPa at 10% compression rate to 63.2 MPa at 25% compression rate, with an increase of only 0.87%, but when the seawater pressure is 115 MPa, the maximum contact stress increases from 117.8 MPa to 119.9 MPa, with an increase of 1.71%, which is about 2 times that of the former.

**Figure 15:**
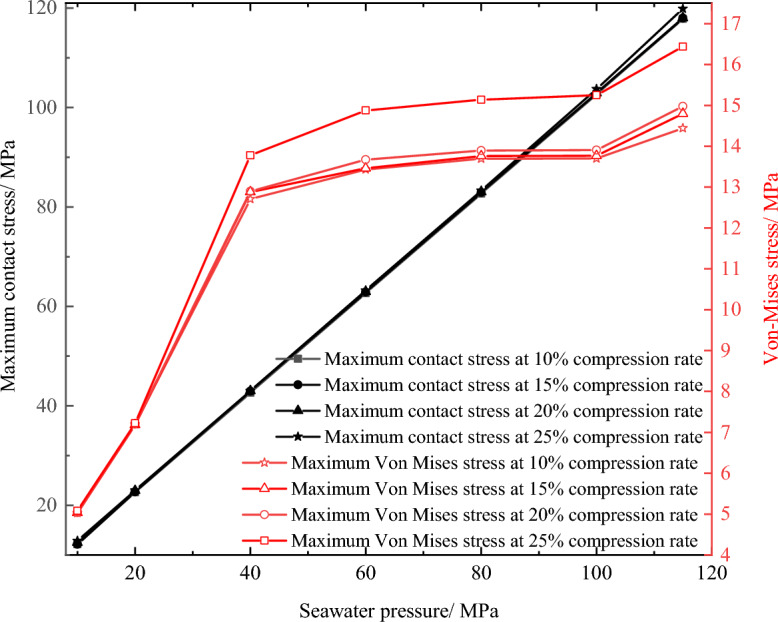
Contact stress and maximum Mises stress of O-shaped rubber at different compression ratios (with a material hardness of 85HA).

In addition, the maximum contact stress of the seal increased approximately linearly with increasing seawater pressure, as shown in Fig. 15. This figure also shows that the maximum Von-Mises stress increased with increasing compression ratio, but this change trend shows a rapid initial increase, followed by a leveling off before continuously increasing (100 ~ 115 MPa stage). It is not difficult to see that the phenomenon of intensifying stress concentration was most obvious in the ultra-high-pressure stage.

Increasing the pre-compression ratio effectively increases the contact stress and thus facilitates establishing the initial seal. However, this increased the maximum Von-Mises stress, which can cause wear and tear as well as permanent deformation of the seal, thus affecting the reliability of the seal. Therefore, when selecting the compression ratio of the seal, the contact stress and Von-Mises stress should be accounted for to ensure that the O-ring undergoes as little permanent deformation as possible while also being put under sufficient contact stress.

Generally, machining errors, assembly errors, and temperature changes can cause changes in the groove size and the initial compression ratio of the O-ring, thus affecting the sealing performance. Specifically, in the design of sealing structure, the machining size and accuracy of sealing surface should be reasonably designed and manufactured, the O-ring compression ratio should be reasonably selected, and the stress concentration should be minimized while still meeting the conditions for maximum contact stress to ensure good sealing performance.

## Experiment in sealing performance

This internal pressure experiment uses a hydraulic pump with rated pressure of 130 MPa, and the experimental medium is water. According to the simulation results, the relevant parameters of the experiment were determined: the compression rate of the seal ring was 10%, the hardness of the material was 85HA, and the experimental pressure was 115 MPa.

According to the “Super-high Pressure Vessel Safety and Technical Supervision Regulations”^25^, The pressure of internal pressure experiment is calculated according to the following formula:

20 $$ p_{{\text{t}}} = \zeta {\kern 1pt} p\frac{{R_{P0.2}^{{}} }}{{R_{P0.2}^{t} }} $$

where *p* is the working pressure of the ultra-high-pressure vessel, i.e.,115 MPa; $\zeta {\kern 1pt}$ is the pressure coefficient of the internal pressure experiment; $R_{p0.2}$ is the lower limit value of yield strength of TC4 material at experimental temperature (MPa);$R_{{_{p0.2} }}^{{\text{t}}}$ is the lower limit value of yield strength of TC4 material at design temperature (MPa).

The maximum working pressure of the sampler is 115 MPa, which belongs to the ultra-high-pressure vessel according to the classification of pressure vessels. the pressure coefficient of internal pressure experiment is taken as 1.1 to 1.25, and the experimental pressure(*p*_t_) is not more than 1.5 *p*. In this paper, based on the safety considerations, we take ζ as 1.12, so the internal pressure experimental pressure is:

21 $$ p_{{\text{t}}} = \zeta {\kern 1pt} {\kern 1pt} p\frac{{R_{P0.2}^{{}} }}{{R_{P0.2}^{t} }} = 1.12 \times 115 = 129\;({\text{MPa}}) $$

### Experimental system

**Figure 16:**
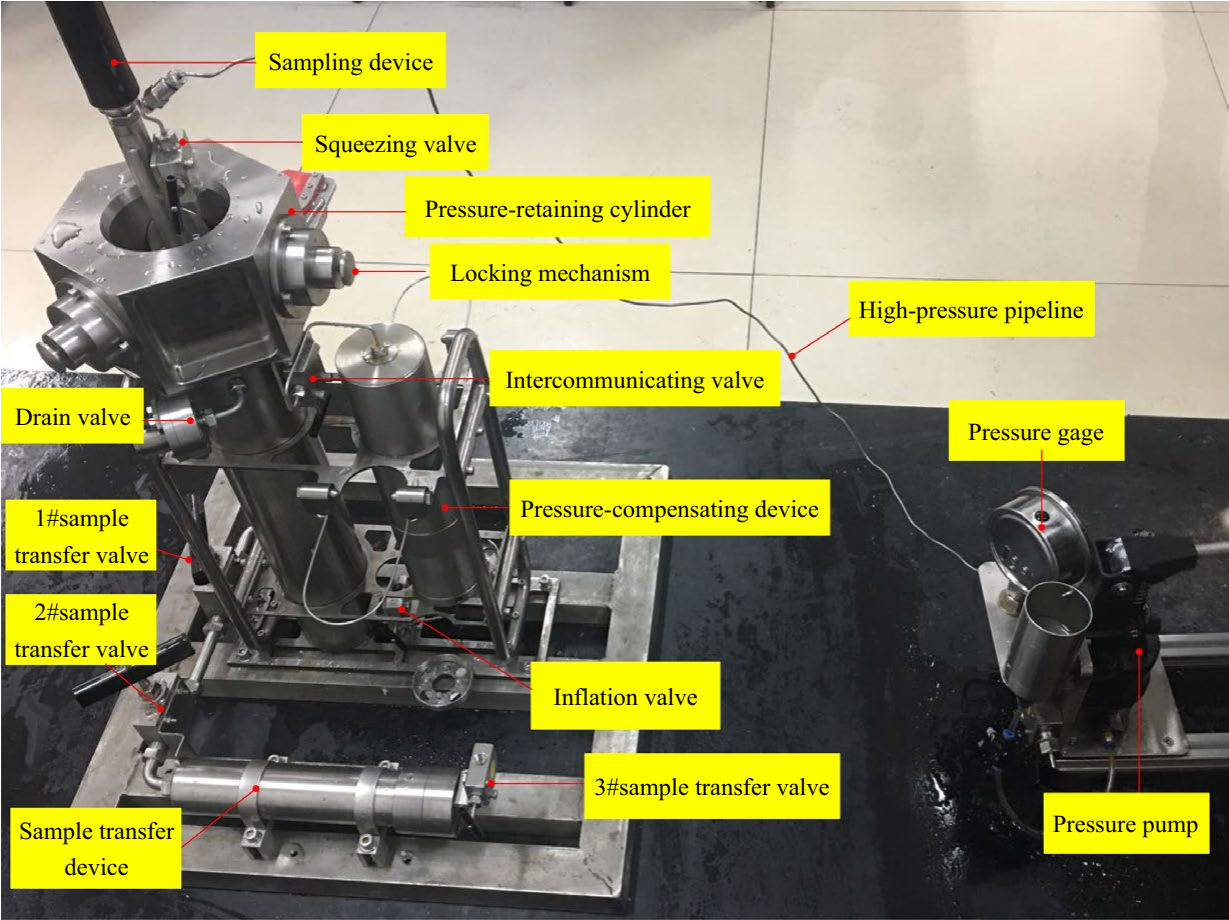
Sealing performance test of the sampler.

An internal pressure experiment of the sampler was conducted in the laboratory to test the pressure resistance and sealing performance. The schematic diagram of the sampler internal pressure experiment is shown in Fig. 16.

### Experimental procedures

1. Add water to the pressure-retaining cylinder of the sampler until water fills the chamber; after confirming that there is no leakage, insert the sampling tube until it is locked, and close the drain valve;
2. Connect the pressurization system to the pressure-retaining cylinder, slowly pressurize the pressure-retaining cylinder by opening the squeeze valve and ensure that the pressure is not less than 13 MPa (10% of the experimental pressure); maintain the pressure for 3 to 5 min; record the pressure data;
3. Continue to pressurize the pressure-retaining cylinder and ensure that the pressure is not less than 65 MPa (50% of the experimental pressure); maintain the pressure for 3 to 5 min; record the data;
4. Next, continue to pressurize and ensure that the pressure is not less than 78 MPa, 91 MPa, 104 MPa, and 115 MPa (gradually increase the pressure to the working pressure by 10% of the specified experimental pressure); hold the pressure at each level for 3 to 5 min; at 115 MPa, close the squeeze valve, filling valve, and 3# sample transfer valve; hold pressure for 120 min; check the connection parts and the connection piping, record the data;
5. Continue to pressurize to the experimental pressure-retaining cylinder to 129 MPa through the squeeze valve, and close the squeeze valve again; hold the pressure for 30 min to ensure that there is no leakage in the connecting pipelines and interfaces; record the data;
6. Open the pressure relief valve, slowly depressurize to the working pressure, hold pressure for 120 min (ensure that the pressure does not drop below 115 MPa); record the experimental data; slowly depressurize based on the pressure differences used in the pressurizing stage; hold each pressure for 3 to 5 min; record the data.

### Experimental results

The experimental results are shown in Table 2. After holding the pressure for 30 min at the experimental pressure of 130 MPa, the pressure-retaining cylinder and sealing structure of the sampler were not damaged and no leakage occurred, which demonstrated that the sampler had sufficient strength for ultra-high pressure conditions. When held at the working pressure (117 MPa) for 4 h, the pressure drop in the pressure-retaining cylinder was about 1.3% to 1.8%, with an average pressure loss of 1.55%. During this period, no sealing failures occurred. Furthermore, the performance index requirement that the pressure-retaining rate be more than or equal to 80% was satisfied. This demonstrated the good pressure resistance and pressure-retaining performance of the sampler, and that the sealing structure of the sampler can guarantee reliable sealing under pressure at the full ocean depth.

**Table 2:** Data from the internal pressure experiment.

No	Experimental pressure/MPa		Pressure-holding time/min	Pressure loss/MPa	Pressure loss/(%)
	Standard value	Actual value			
1	13	14	5	0.5	3.6
2	65	66	5	1.5	2.3
3	78	79	5	1.0	1.3
4	91	92	5	0.75	0.8
5	104	105	5	1.25	1.2
6	115	117	120	2.0	1.8
7	129	130	30	1.0	0.7
8	115	117	120	1.5	1.3
9	104	105	5	0.75	0.7
10	91	92	5	0.5	0.5
11	78	79	5	0.5	0.7
12	65	66	5	0.25	0.6
13	13	14	5	0.25	1.8

## Conclusion

1. This study on the sealing performance of the sampler under ultra-high-pressure conditions elucidated the mechanism of a self-tightening O-ring seal. The precondition necessary for forming the self-tightening seal was obtained through calculations, i.e., the coefficient of friction on the seal surface must be less than or equal to 0.25. The principle of the sealing process under ultra-high pressures was explored, and the influences of seawater pressure, compression rate and material harness were studied, which provides a scientific basis for determining the sealing structure and structural parameters of sediment sampler under ultra-high pressure. The main conclusions are as follows:
  - The maximum contact stress on the O-ring seal increased approximately linearly with increasing seawater pressure, but in each case, the maximum contact stress was greater than the corresponding seawater pressure. However, the maximum Von-Mises stress did not change linearly with seawater pressure, and instead increased rapidly at first, then leveled off, and then continuously increase in the 100 ~ 115 MPa range. Under the seawater pressure of 115 MPa, the maximum Von-Mises stress of the O-ring was 14.44 MPa, which was smaller than the extension strength of the O-ring and was well withing acceptable parameters.
  - Increasing the compression rate can effectively increase the contact stress, which facilitates the establishment of the initial seal. However, increasing the compression rate also increases the maximum Von-Mises stress, as was observed here, especially in the ultra-high-pressure stage when the stress concentration intensified.
  - With increasing material hardness of the O-ring, the maximum principal strain gradually decreased, but the maximum Mises stress increased. This was observed in the compression rate range from 10 to 25%. At higher compression rates the contact stress was higher, but the maximum Mises stress increased as well. By comparing O-rings with different hardness values, it was observed that the increased material hardness had a very significant impact on the initial contact pressure.
2. The pressure-retaining performance experiment showed that, over 4 h, the average pressure loss under the experimental pressure (117 MPa) was only 1.55%. Furthermore, there was no occurrence of seal failure, which verified that the calculation model was correct and showed that the sealing structure of the sampler can guarantee reliable sealing under pressures found at the full ocean depth.
3. This paper only analyzes the influence of three main factors on the sealing performance. In fact, the sampler works in the complicated environment under seawater with ultra-high pressure, and the sealing performance may also be affected by seawater temperature, sealing surface machining accuracy, alternating loads, etc. The influence of these factors on the sealing performance should also be considered in the future. At the same time, future studies should be combined with experimental research to build a more perfect numerical model, thus providing a theoretical basis for the design of the sealing structure of deep-sea equipment.

## Data Availability

The datasets used and analysed during the current study available from the corresponding author on reasonable request.

## References

[1] Ramirez-Llodra E (2010). Deep, diverse and definitely different: Unique attributes of the world’s largest ecosystem. Biogeosciences.

[2] Shuhua C, Chen G, Zhen L, Tianlin W (2020). Numerical analysis of underwater glider sealing structure in deep sea environment. Lubr. Eng..

[3] Qiao LN, Keller C, Zencker U, Vo¨lzke H (2019). Three-dimensional finite element analysis of O-ring metal seals considering varying material properties and different sealdiameters. Inter. J. Press. Vessels Pip..

[4] Davies P, Le Gac PY, Ciausu V, Gallois H (2020). Testing of nitrile rubber joints for a deep submergence vehicle. Polym. Test..

[5] Chen GD, Haiser H (2000). Finite element mechanical analysis of O-ring seal. Mech. Sci. Technol..

[6] Zhou ZH, Zhang KL, Li J, Xu TL (2006). Finite element analysis of stress and contact pressure on the rubber sealing O-ring. Lubr. Eng..

[7] Nikas GK (2004). Theoretical study of solid back-up rings for elastomeric seals in hydraulic actuators. Tribol. Int..

[8] Abouel-Kasem A (2006). Numerical analysis of leakage rate for the selection of elastomeric sealing materials. Sealing Technol..

[9] Nikas GK (2004). Theoretical study of solid back-up rings for elastomeric seals in hydraulic actuators. Tribol Int..

[10] Salant RF, Maser N, Yang B (2007). Numerical model of a reciprocating hydraulic rod seal. ASME J. Tribol..

[11] Patir N, Cheng HS (1979). Application of average flow model to lubrication between rough sliding surfaces. J. Lubr. Technol..

[12] Hirano, F. & Kaneta, M. Experimental investigation of friction and sealing characteristics of flexible seals for reciprocating motion, in *Proceedings of the 5th International Conference on Fluid sealing*, 33–48 (1971).

[13] Aston, M., Fletcher, W. & Morrell, S. H. Sealing force of rubber seals and its measurement, in *Proceedings of the 4th International Conference on Fluid sealing* 64–75 (1969).

[14] Hirano, F, & Kaneta, M. Theoretical investigation of friction and sealing characteristics of flexible seals for reciprocating motion, in *Proceedings of the 5th International Conference on Fluid sealing* .17–32 (1971).

[15] Nikas GK (2003). Transient elastohydrodynamic lubrication of rectangular elastomeric seals for linear hydraulic actuators. Proc. Inst. Mech. Eng. Part J: J. Eng.Tribol..

[16] Wang, B. Q., Peng, X. D. & Meng, X. K.. Elastohydrodynamic lubrication characteristics of an O-ring hydraulic rod seal during transient peration. *UK: IET Conference Publications*.348–353 (2018).

[17] Rivlin, R. S. & Saunders, D.W. Large elastic deformations of isotropic materials//Collected papers of RS Rivlin. *Springer, New York, NY:* 157–194 (1997).

[18] Marckmann G, Verron E (2006). Comparison of hyperelastic models for rubber-like materials. Rubber Chem. Technol..

[19] Shi S, Yang J (1999). The penalty function method is used to analyze the large deformation of a class of incompressible rubber. J. Appl. Basic Eng. Sci..

[20] Guang T, Wang D (1994). Seal user manual.

[21] Afrusinco. Rubber seal. Liu Yaozu, Trans. *China Machine Press*, (1983).

[22] Yongquan G (1990). Fluid dynamic seal.

[23] Wensel RG (1988). O-ring seal seal studies for space and space shuttle solid rocket booster joints. Can .Aeronaut. Space J..

[24] Sivakumar V, Palaninathan R (2012). FE analysis of contact pressure prediction on o-rings used in solid rocket booster segment joints. Int. J. Sci. Eng. Appl.

[25] "Safety technical supervision regulations for ultra high pressure vessels." TSG R0002-2005. 2005-11-08.

